# Simultaneous Partial Nitrification and Denitrification Maintained in Membrane Bioreactor for Nitrogen Removal and Hydrogen Autotrophic Denitrification for Further Treatment

**DOI:** 10.3390/membranes11120911

**Published:** 2021-11-23

**Authors:** Kun Dong, Xinghui Feng, Wubin Wang, Yuchao Chen, Wei Hu, Haixiang Li, Dunqiu Wang

**Affiliations:** College of Environmental Science and Engineering, Guilin University of Technology, 319 Yanshan Street, Guilin 541006, China; 2020005@glut.edu.cn (K.D.); xinghfeng@glut.edu.cn (X.F.); wangwubin2021@163.com (W.W.); 1020180208@glut.edu.cn (Y.C.); huwei5018@163.com (W.H.)

**Keywords:** partial nitrification-denitrification, hydrogen autotrophic denitrification, MBR-MBfR

## Abstract

Low C/N wastewater results from a wide range of factors that significantly harm the environment. They include insufficient carbon sources, low denitrification efficiency, and ${\mathrm{NH}}_{4}^{+}$-N concentrations in low C/N wastewater that are too high to be treated. In this research, the membrane biofilm reactor and hydrogen-based membrane biofilm reactor (MBR-MBfR) were optimized and regulated under different operating parameters: the simulated domestic sewage with low C/N was domesticated and the domestic sewage was then denitrified. The results of the MBR-MBfR experiments indicated that a C/N ratio of two was suitable for ${\mathrm{NH}}_{4}^{+}$-N, ${\mathrm{NO}}_{2}^{-}$-N, ${\mathrm{NO}}_{3}^{-}$-N, and chemical oxygen demand (COD) removal in partial nitrification-denitrification (PN-D) and hydrogen autotrophic denitrification for further treatment. The steady state for domestic wastewater was reached when the MBR-MBfR in the experimental conditions of HRT = 15 h, SRT = 20 d, 0.04 Mpa for H_2_ pressure in MBfR, 0.4–0.8 mg/L DO in MBR, MLSS = 2500 mg/L(MBR) and 2800 mg/L(MBfR), and effluent concentrations of ${\mathrm{NH}}_{4}^{+}$-N, ${\mathrm{NO}}_{3}^{-}$-N, and ${\mathrm{NO}}_{2}^{-}$-N were 4.3 ± 0.5, 1.95 ± 0.04, and 2.05 ± 0.15 mg/L, respectively. High-throughput sequencing results revealed the following: (1) The genus *Nitrosomonas* as the ammonia oxidizing bacteria (AOB) and *Denitratisoma* as potential denitrifiers were simultaneously enriched in the MBR; (2) at the genus level, *Meiothermus,* *Lentimicrobium, Thauera,* *Hydrogenophaga*, and *Desulfotomaculum* played a dominant role in leading to ${\mathrm{NO}}_{3}^{-}$-N and ${\mathrm{NO}}_{2}^{-}$-N removal in the MBfR.

## 1. Introduction

Conventional biological nitrogen removal (BNR) includes ammonification, nitrification, and denitrification. This type of approach is considered to be a good choice for reducing nitrogenous compounds in wastewater treatment because it is economic, effective, easy to operate, and results in no secondary pollution [1,2]. However, for low C/N (chemical oxygen demand (COD)/ammonia nitrogen, ${\mathrm{NH}}_{4}^{+}$-N) wastewater, the BNR imposes stringent restrictions on the insufficient carbon source and results in incomplete nitrogen removal, which in turn requires external organic carbon sources, high operating costs, and high aeration-associated energy consumption for nitrification [3,4,5].Partial nitrification has been regarded as a sound self-sustaining biological nitrogen removal process because it can reduce aeration energy by 25%, reduce carbon dioxide emissions and sludge production, decrease organic carbon requirement by 40–100%, and reduce biomass production by 300% [1,6]. According to Equations (1) and (2), the effectiveness of partial nitrification was closely determined by the concentration of influent ${\mathrm{NH}}_{4}^{+}$ and the precise control of dissolved oxygen (DO) concentration. In addition, ${\mathrm{NO}}_{2}^{-}$-N did not coexist with ${\mathrm{NH}}_{4}^{+}$-N in most of the wastewater (usually needed in situ conversion to initialize the partial nitrification process), but ${\mathrm{NO}}_{3}^{-}$-N and COD were frequently found in water and wastewater:

(1) $${\mathrm{NH}}_{4}^{+}\;+\;1.5O_{2}\to {\mathrm{NO}}_{2}^{-}\;+\;2H^{+}\;+\;H_{2}O,$$

(2) $${\mathrm{NO}}_{2}^{-}\;+\;1.5O_{2}\to {\mathrm{NO}}_{3}^{-}$$

Various forms of integrated techniques were implemented in the past few years to improve the ability of total nitrogen (TN) removal and to treat low C/N wastewater; novel and cost-effective partial nitrification-based BNR processes have been put forward, including partial nitrification-denitrification (PN-D), partial nitrification-simultaneous anammox and denitrification (PN-SAD), and anaerobic ammonium oxidation (Anammox). PN-D may represent a good alternative compared with conventional nitrification-denitrification because the process uses nitrite nitrogen as an electron acceptor and organic matter as an electron donor. It has the advantages of lower yields of sludge, being energy saving (low aeration consumption), reducing the carbon source, and being suited for low C/N wastewater [7]. Partial nitrification combined with denitrification achieves excellent nitrite accumulation through the accumulation of ammonia-oxidizing bacteria (AOB) and the inhibition of nitrite-oxidizing bacteria (NOB) in the reactors [8].

Considerable effort was made to control the operating conditions. However, it was still hard to avoid the effluent ${\mathrm{NO}}_{2}^{-}$-N concentration, the ${\mathrm{NO}}_{3}^{-}$-N concentration, and COD exceeding the stringent discharge standard all the time. Therefore, further improvements of the ${\mathrm{NO}}_{2}^{-}$-N concentration, ${\mathrm{NO}}_{3}^{-}$-N concentration, and COD were necessary. Furthermore, low DO aeration and ${\mathrm{NH}}_{4}^{+}$-N residues were conducive to partial nitrification stability [4]. There were many factors affecting partial nitrification, including but not limited to DO, temperature, pH, free ammonia (FA), and free nitrous acid (FNA). The oxygen saturation concentration was 0.3 mg/L and 1.1 mg/L for the ammonia oxidation reaction and nitrite oxidation reactions, respectively, and the low DO (less than 0.5 mg/L) benefitted AOB and inhibited the NOB [9]. Li et al. [10] reported that the optimal temperature for AOB and anaerobic ammonium oxidizing bacteria (AnAOB) for partial nitrification was 35 °C. A suitable pH for the partial nitrification process was 6.5–8.5. When the pH was higher than 8.5, the partial nitrification was inhibited and the alkaline consumption was increased [11]. Soliman et al. [12] reported that the inhibition limit for NOB was 0.1–1.0 mg N/L and the inhibition limit for AOB was 10–150 mg N/L of FA. The start of the partial nitrification process was restricted by the aforementioned conditions and its industrial applications were severely hindered. Therefore, to the method of quickly starting and maintaining a stable partial nitrification reaction remains extremely challenging.

At present, the widely reported partial nitrification processes are built into sequential batch reactors (SBR). However, SBR reactors are constrained by complicated control and poor stability performance [13]. Membrane bioreactors (MBRs) are alternatives to be applied in the PN-D process. They can avoid biomass wash-out and increase biomass retention, which allows the reactor to operate at a high biomass concentration, therefore improving the stability of the PN-D process. However, poor control of oxygen and other influencing factors lead to the production of ${\mathrm{NO}}_{2}^{-}$-N and ${\mathrm{NO}}_{3}^{-}$-N, as shown in Equations (1) and (2). This can lead to the quality of the effluent exceeding the wastewater disposal standards if no further treatment is applied.

The hydrogen-based membrane biofilm reactor (MBfR), an emerging biodegradation technology based on a hydrogenotrophic reduction process, efficiently removes nitrite and nitrate from wastewater. In the MBfR, pressurized H_2_ is supplied to the lumen of the hollow fiber membrane (HFM). The gas diffuses through the HFM wall through nanopores in the autotrophic biofilm formed on the outer HFM surface. Here, nitrate and nitrite diffuse from water into biofilm being reduced [14,15]. The advantages of this hydrogenotrophic denitrification compared with conventional heterotrophic denitrification technology include the utilization of nontoxic and inexpensive H_2_ as electron donors, no requirement for the addition of external organic carbon, a small footprint, relatively low cost, and low production of biological sludge [16,17]. Promising outcomes have been reported with MBfR for treating nitrate- and nitrite-contaminated water for research and commercial applications. For example, Park et al. [18] applied an MBfR to treat high-strength wastewater containing 50 mg/L ${\mathrm{NO}}_{3}^{-}$-N without supplying any source of organic carbon and a maximum nitrate removal rate of 0.1 g ${\mathrm{NO}}_{3}^{-}$-N/(m^2^d) was achieved. In addition, heterotrophs are usually also present in MBfR, which may bring further removal of COD and contribute to the denitrification in a heterotrophic pathway if COD is present in the influent. Therefore, an MBfR could be ideally suited for the treatment of nitrate and nitrite byproducts from processes of partial nitrification and excessive COD.

In this study, strategies to initiate start-up NOB suppression and to adapt the partial nitrification process to a hydrogenotrophic denitrification process were proposed. The objectives of this study were as follows: (1) to couple partial-nitrification and denitrification in MBR (MLSS = 2500 mg/L, 0.4–0.8 mg/L DO, reactor temperature was 35 °C) and use a small amount of carbon source to achieve high-efficiency nitrogen removal through a denitrification process; (2) to experimentally take advantage of MBfR (H_2_ pressure was 0.04 MPa and the pH value around 7.5) to quickly remove TN (nitrate and nitrite) and low concentrations of nitrous; and (3) to explore the most suitable operating parameters for low C/N wastewater when MBR-MBfR (in the experimental conditions of HRT = 15 h, SRT = 20 d) was employed for domestic wastewater treatment.

## 2. Materials and Methods

### 2.1. Influent

The reactor was operated with both synthetic wastewater and domestic wastewater. The synthetic wastewater used in this study was composed of substrates and trace elements as influent. Ammonium was provided in the form of NH_4_Cl and added as required. The pH in the reactor was maintained automatically at 7.5 ± 0.5 by addition of 0.1 M HCl and 0.1 M NaOH. An aeration device was set at the water pipe to control the concentration of DO between 0.4 and 0.8 mg/L. Peristaltic pumps (BT101L-DG-1, Lead Fluid, Baoding, China) were used to control the influent and the effluent feed rate. The synthetic domestication water was adapted from an earlier study and comprised the following components per liter [19]: 1 g NaHCO_3_, 0.1 g CaCl_2_.2H_2_O, 0.05 g KH_2_PO_4_, 0.05 g Na_2_SO_3_, and 1 mL of a stock solution containing trace elements. The trace element stock solution contained (per liter): 5 g MgSO_4_·7H_2_O, 0.1 g ZnSO_4_.7H_2_O, 6 g FeCl_2_·4H_2_O, 0.1 g H_3_BO_3_, 0.88 g CoCl_2_·6H_2_O, 0.5 g MnCl_2_.4H_2_O, 0.036 g NiCl_2_·6H_2_O, and 0.035 g CuCl_2_·2H_2_O. The domestic wastewater was obtained from the effluent of a primary settling tank in a domestic wastewater treatment plant (WWTP) from Guilin University of Technology. The domestic wastewater conditions were as follows (similar to the operational conditions of the batch experiment in phase III with various C/N ratios in the MBR): COD, 140–160 mg/L; ${\mathrm{NH}}_{4}^{+}$-N, 80–100 mg/L; ${\mathrm{NO}}_{3}^{-}$-N, 5–10 mg/L; ${\mathrm{NO}}_{2}^{-}$-N, 0 mg/L; C/N ratio, 2.

### 2.2. Reactor Configuration

**Figure 1:**
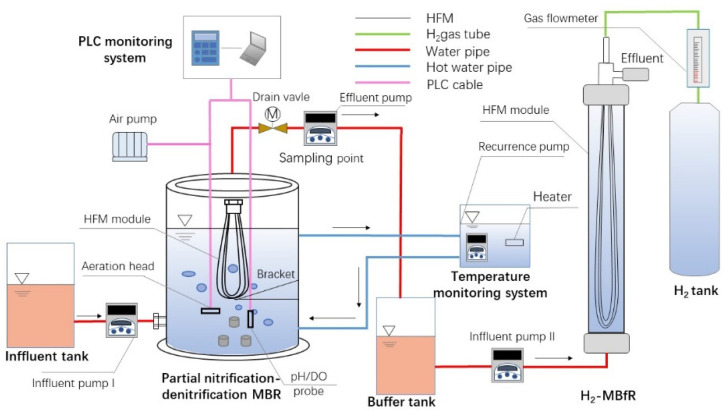
Schematic representation of the MBR-MBfR set-up.

A scheme of the MBR-MBfR used in this study is shown in Figure 1 and the physical characteristics of the reactors are listed in Table 1. The MBR set-up was made of plexiglass and consisted of four parts: (I) an inner MBR unit used for culturing PN-D sludge; (II) a thermostatic jacket filled with hot water to maintain a fixed temperature of 35 °C for PN-D; (III) a hollow fiber membrane (HFM) module, in which the HFM was made of commercially available PVC; and (IV) a programmable logic controller (PLC) system for monitoring the pH and for controlling the concentration of DO. Notably, the effluent of MBR was realized by the operation of a pump, in which the outlet at the top of the MBR was connected to a buffer tank placed lower than the MBR for further treatment.

The start-up processes for MBR and MBfR were conducted under different operating conditions. The startup operation for MBR was divided into two periods. The MBR was inoculated with 500 mL of partial nitrification bacterial sludge: an initially mixed liquor suspended solids (MLSS) of 2500 mg/L from a stable operating partial nitrification reactor in the first period and 500 mL of denitrification bacterial sludge (MLSS of 2500 mg/L) from a stable operating denitrification reactor in the second period. The start-up phase began using synthetic wastewater containing ${\mathrm{NH}}_{4}^{+}$-N under the conditions of low DO (0.4–0.8 mg/L). The SRT of the MBR was 20d. The startup operation for MBfR was HRT = 10 h (shortened to 5 h in the post-start experiment), MLSS = 2800 mg/L, and the SRT of the MBfR was 20d.

The medium consisted of tap water with the following components added: 1.386 g Na_2_PO_4_, 0.849 g KH_2_PO_4_, 0.05 g MgSO_4_·7H_2_O, and 0.025 g (NH_4_)_2_SO_4_ per liter. NaNO_3_ and NaHCO_3_ were used as inorganic nitrogen and carbon sources for the rapid growth of hydrogenotrophic microorganisms. A mixture of KH_2_PO_4_ 0.128 g/L and Na_2_HPO_4_ 0.434 g/L was used as the phosphate buffer to keep the pH value of the MBfR around 7.5 [20]. The hollow-fiber membranes were made from microporous polyethylene with a thin polyurethane core (Watercode, Guangzhou, China). The total membrane active surface area of the MBfR was 0.28 m^2^. The MBfR system consisted of an HFM module with 65 HFMs located inside of a vertical plexiglass cylindrical shell and an ultrapure H_2_ tank for supplying pressurized H_2_ to the HFM module. The MBfR module was sealed using waterproof epoxy glue. The fiber was connected to a hydrogen-supplying manifold supplied at the top end and sealed individually at the bottom end. Smaller pore size HFMs made of PVC with a pore size of 0.02 μm were used in the MBfR to deliver bubble-less H_2_ through the HFM wall. The H_2_ pressure was 0.04 MPa, while the pressure was also adjusted in the range of 0.02–0.06 MPa to evaluate the effect of H_2_ pressure on nitrogen removal performance.

### 2.3. Samping and Analytical Methods

The operating performance of the reactors was evaluated by analyzing influent and effluent samples on a daily basis. Samples were subsequently filtered through a 0.22 μm membrane filter. Influent and effluent samples were collected daily for both MBR and MBfR to analyze the concentration of ${\mathrm{NH}}_{4}^{+}$-N, ${\mathrm{NO}}_{3}^{-}$-N, and TN according to a standard method provided by the American Public Health Association (APHA). ${\mathrm{NO}}_{2}^{-}$-N concentrations were determined using a colorimetric assay based on sulfanilamide and *N*-(1-naphthyl) ethylenediamine dihydrochloride. The absorbance was measured at 540 nm in a spectrophotometer (UV6100, METASH, Shanghai, China). The pH and DO were monitored in situ via a PLC system that was connected with pH and DO probes.

The biomass samples were sent out for microbial structure analysis at Novogene Co., Ltd. (Beijing, China). The relative abundance of partial nitrification bacteria, denitrification bacteria, and hydrogen autotrophic denitrifying bacteria were determined by high-throughput sequencing analysis. The V4-V5 hypervariable region of the 16s RNA gene was amplified by polymerase chain reaction (PCR). The amplified PCR used the bacterial primers 515F(5′-GTGCCAGCMGCCGCGGTAA-3′) and 907R(5′-CCGTCAATTCCTTTGAGTTT-3′). The operational taxonomic units (OTUs) of the representative sequences were annotated with the Ribosomal Database Project (RDP) classifier method and the Greengene database [21] for species annotation analysis (the threshold value was set to 0.8–1). The sequence number of each sample was normalized and the trimmed sequences were grouped into OTUs using 97% identity thresholds. [22]. Kingdom, phylum, class, order, family, genus, and species were used to analyze the community composition of each sample. Taxonomic classification at the genus level was performed using the RDP algorithm to classify the representative sequences from each OTU.

## 3. Results and Discussion

### 3.1. Start-Up and Experimental Strategy of MBR

The startup operation for MBR was divided into two periods (shown in Section 2.2). In the first period, which consists of the demand for partial nitrification bacteria, the incubator was continuously fed by the peristaltic pump with an increasing nitrogen load (gradually increasing the ${\mathrm{NH}}_{4}^{+}$-N concentration and decreasing the hydraulic retention time (HRT); the detailed operating conditions during the start-up process are shown in Table 2). After 30 days of the start-up phase for the partial nitrification operation, more than 96% removal of ${\mathrm{NH}}_{4}^{+}$-N was achieved under a hydraulic retention time (HRT) of 10 h and the concentration of reactor ${\mathrm{NO}}_{2}^{-}$-N rose to 102 mg/L. This was accompanied by a small amount of ${\mathrm{NO}}_{3}^{-}$-N generation (10 mg/L). The start-up and stability of the partial nitrification process were successful under these conditions.

After 31 days, the carbon source was added to the MBR relative to the phase (showed in Table 3) and 500 mL of denitrification bacteria sludge was inoculated in the MBR. A batch experiment with various C/N ratios in the range 0.5–3 was performed in the MBR to see how the performance of denitrification was affected and to promote the stability and acclimatization of denitrification with the co-existence of COD and nitrogen. This was conducted because a certain amount of COD is always present in domestic wastewater. The existence of COD promoted the denitrification process to a certain extent. At this stage, denitrifying bacteria had a certain effect on the removal of COD, ${\mathrm{NO}}_{3}^{-}$-N, and ${\mathrm{NO}}_{2}^{-}$-N, but they could not be completely removed. The operational conditions during this batch experiment are shown in Table 3. The nitrogen concentration in the influent was fixed with a concentration of ${\mathrm{NH}}_{4}^{+}$-N of 80 mg/L, while the influent C/N was increased in a stepwise manner by adding the required volume of white sugar.

**Figure 2:**
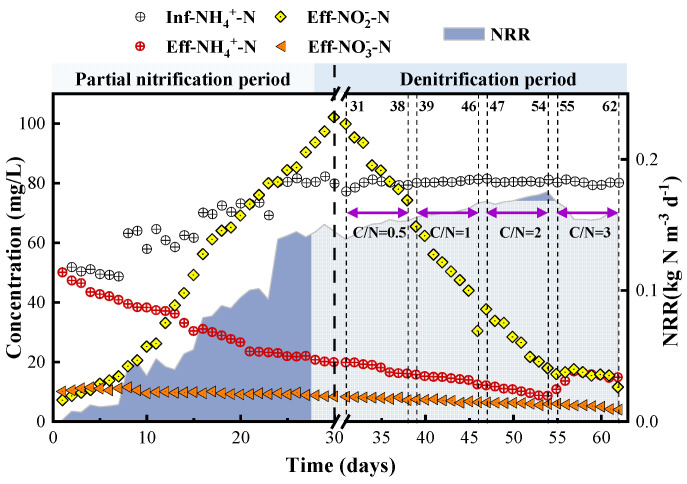
${\mathrm{NH}}_{4}^{+}$-N, ${\mathrm{NO}}_{2}^{-}$-N, and ${\mathrm{NO}}_{3}^{-}$-N in the effluent and ammonia-nitrogen removal rate (NRR) of the start-up of the MBR.

**Figure 3:**
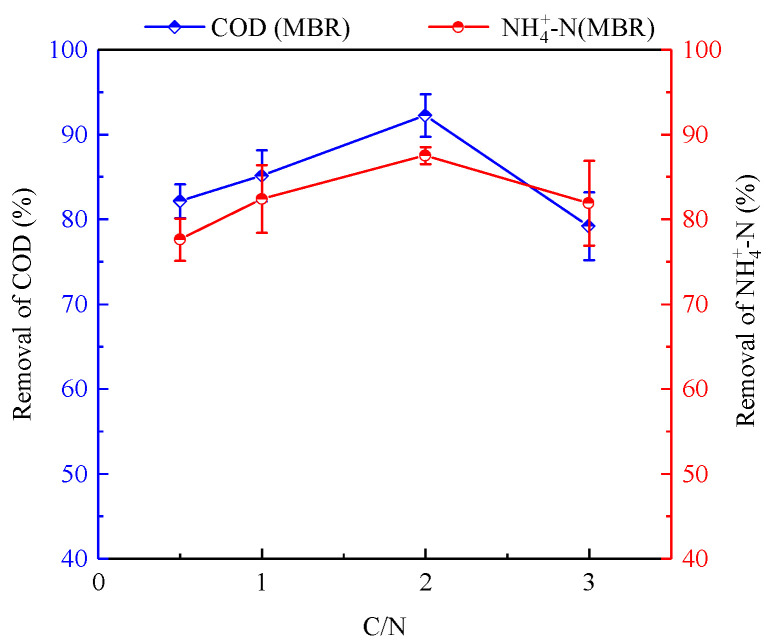
${\mathrm{NH}}_{4}^{+}$-N and COD removal in the MBR during batch experiments in the denitrification period.

An excessively high concentration of organic carbon may therefore significantly suppress the removal of nitrogen via denitrification. The effect of COD on nitrogen and COD removal was evaluated to determine the effectiveness of the denitrification period of MBR. A preliminary batch test with various C/N ratios in the range of 0.5–3 was performed in the MBR after the start-up and steady state of MBR without adding an organic carbon source. The performance of ${\mathrm{NH}}_{4}^{+}$-N and COD removal for the MBR in the denitrification period is shown in Figure 2. Negligible change of COD removal, which was around 13.06%, was found when the C/N ratio was varied in the range of 0.5–3. Notably, the highest ${\mathrm{NH}}_{4}^{+}$-N removal of 87.59% was obtained at a C/N ratio of two, while a dramatic decrease was found when the C/N ratio was higher than two. As shown in Figure 3, 9.9% and 5.2% higher values of ${\mathrm{NH}}_{4}^{+}$-N removal at a C/N ratio of two were observed than those obtained at ratios of 0.5 and 1. This might be attributable to the higher contribution of ${\mathrm{NH}}_{4}^{+}$-N removal through the heterotrophic denitrification pathway with COD concentration in the influent (C/N ratio of two). When the C/N ratio was 0.5 and the influent COD concentration decreased to 40 mg/L, the COD content was low, and the lack of carbon source led to incomplete denitrification. The effluent ${\mathrm{NO}}_{2}^{-}$-N concentration decreased and the COD removal rate decreased. As shown in Figure 2, a C/N ratio of two was suitable for ${\mathrm{NH}}_{4}^{+}$-N, ${\mathrm{NO}}_{2}^{-}$-N, ${\mathrm{NO}}_{3}^{-}$-N, and COD removal and resulted in no apparent inhibition of denitrification activity. However, around 10 mg/L of ${\mathrm{NO}}_{3}^{-}$-N (shown in Figure 2) was detected in the effluent when the C/N ratio in the influent was two in the MBR. When the C/N ratio was three, the activated sludge system in the reactor was a complex multi-bacteria coexistence system. With the increase of C/N in the domestic wastewater, the activity of heterotrophic bacteria and denitrifiers in the reactor increased. The change in the composition of the influent matrix made PN need an adaptation process, but with the increase of organic matter, AOB and denitrifying bacteria also had a dynamic change until a new balance occurred. Increasing organic matter promoted the growth of denitrifiers. Organics promote the growth of denitrifiers, while these denitrifiers or heterotrophs would consume more oxygen, which is necessary for AOB growth, inhibiting the growth of AOB involved in partial nitrification and compete with AOB for living space and substrate [23], leading to an increase in the concentration of ${\mathrm{NH}}_{4}^{+}$-N in the effluent. Too much carbon inhibited the growth of the AOB participating in the partial nitrification. This increased the effluent ${\mathrm{NH}}_{4}^{+}$-N concentration at this phase (C/N ratio of three) and the partial nitrification product ${\mathrm{NO}}_{2}^{-}$-N was used by heterotrophic denitrifying bacteria to produce PN-D. This reduced the nitrite nitrogen concentration and led to an increase of sludge produced by subsequent denitrification. Therefore, a further sufficient treatment of nitrate was required.

### 3.2. Start-Up and Experimental Strategy of MBfR

Different synthetic wastewater containing 10 mg/L of ${\mathrm{NO}}_{3}^{-}$-N was used for the MBfR start-up. The inoculation seeding sludge of hydrogenotrophic bacteria was collected from a lab-scale denitrifying MBfR in our laboratory. The MBfR start-up procedure was like the procedure for the MBR, which was to continuously feed the synthetic influent with increasing loading of nitrate. After successful start-up, the MBfR was able to reach a ${\mathrm{NO}}_{3}^{-}$-N removal of more than 98% at an HRT of 10 h with an influent ${\mathrm{NO}}_{3}^{-}$-N concentration of 10 mg/L. Previous studies proved that the H_2_ supplying pressure and influent nitrate loading were the two key operational factors that affected the nitrate removal performance in MBfR [24,25].

In this study, two series of experiments were conducted to investigate the performance of nitrate removal in the MBfR. These included looking at the H_2_ supplying pressure and influent ${\mathrm{NO}}_{3}^{-}$-N concentration. A H_2_ supplying pressure in the range of 0.02–0.08 MPa has been acknowledged to be preferable for use in most MBfRs [26]. In the H_2_ series, three pressures of 0.02, 0.04, and 0.06 MPa were involved to discover the optimal H_2_ pressure for our reactor, while the influent ${\mathrm{NO}}_{3}^{-}$-N concentration was fixed at 10 mg/L as the H_2_ pressure varied. Most natural water in China usually has a certain amount of nitrate (e.g., 10 mg/L of ${\mathrm{NO}}_{3}^{-}$-N was detected in tap water used to make synthetic medium in our lab). Therefore, we added 10 mg/L ${\mathrm{NO}}_{3}^{-}$-N during the H_2_ series. For the ${\mathrm{NO}}_{3}^{-}$-N series, the influent contained 10, 20, and 30 mg/L of ${\mathrm{NO}}_{3}^{-}$-N to discover the potential ability of nitrate removal in the MBfR and to evaluate the capability to encounter fluctuations of influent. The H_2_ pressure was fixed at 0.04 MPa. The HRT was maintained at 5 h for all the experiments in the two series.

**Figure 4:**
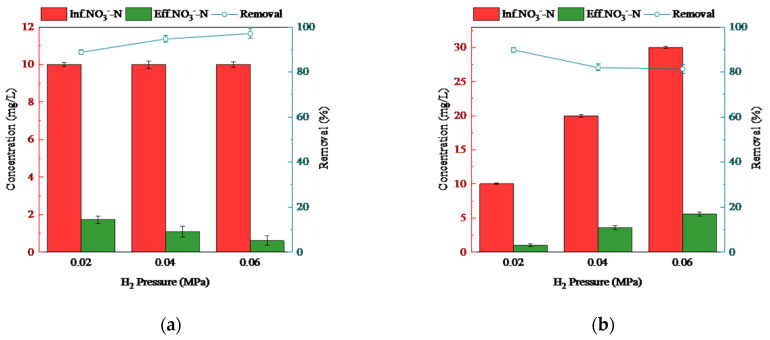
Effects of H_2_ pressure and influent ${\mathrm{NO}}_{3}^{-}$-N concentration on ${\mathrm{NO}}_{3}^{-}$-N concentration and removal in the effluent. (**a**) H_2_ pressure: 0.02, 0.04, and 0.06 MPa; (**b**) influent ${\mathrm{NO}}_{3}^{-}$-N concentration: 10, 20, and 30 mg/L.

The MBfR was used to handle the left-over nitrate produced in the PN-D process. We assessed the effects of two key factors on the performance of nitrate removal, namely, H_2_ pressure and influent ${\mathrm{NO}}_{3}^{-}$-N concentration, to discover the capability of the MBfR to remove nitrate. The results are shown in Figure 4. An apparent increase of nitrate removal was observed from 96% to 99%, when the H_2_ pressure increased from 0.02 MPa to 0.04 MPa, compared with an increase from 0.04 MPa to 0.06 MPa (less than 1%) as shown in Figure 4a. MBfR was therefore already able to efficiently remove nitrate from the influent when the H_2_ supplying pressure was set at 0.04 MPa. It was not necessary to use a higher supplying pressure, which helped with safety and preserved the life of the membranes. The influent ${\mathrm{NO}}_{3}^{-}$-N concentration was varied at 10, 20, and 30 mg/L and the effect on nitrate removal is shown in Figure 4b. The influent ${\mathrm{NO}}_{3}^{-}$-N concentration in this range covered the nitrate produced from PN-D MBR plus the fluctuation of nitrate concentration in the tap water used for synthetic wastewater or domestic wastewater. The highest effluent ${\mathrm{NO}}_{3}^{-}$-N concentration of 3.2 mg/L was found in all three phases for drinking water when the influent ${\mathrm{NO}}_{3}^{-}$-N concentration was 30 mg/L. A stepwise decrease of nitrate removal was found as the influent concentration of ${\mathrm{NO}}_{3}^{-}$-N increased.

### 3.3. Effects of the MBR-MBfR on Nitrogen Compound Removal at C/N = 1.5–2.5

The domestic wastewater was tested in the integrated MBR-MBfR to verify the application of the system. The domestic wastewater was obtained from the effluent of a primary settling tank in a wastewater treatment plant. Of note, 120–160 mg/L COD was added proportionally to the influent at the appropriate concentration. This addition was done to validate the PN-D process to biodegrade nitrogen and COD, and to verify the optimum C/N ratio of PN-D and hydrogen autotrophic denitrification for further treatment in the MBR-MBfR. The domestic wastewater was also fed continuously at an HRT of 15 h for both MBR and MBfR.

Extensive experiments in three phases were conducted under various C/N conditions to investigate the behaviors in both the MBR and MBfR and how the nitrogen and COD removal performed in each reactor. This was achieved based on the PN-D process and adding simulated wastewater at different C/N ratios. Each phase was operated long enough to reach steady state. The HRT of the MBR and MBfR was set to 10 h and 5 h, respectively, which resulted in a total HRT of 15 h for the integrated MBR-MBfR system. The operational conditions under different phases are summarized in Table 4. A C/N ratio of two was still suitable for the remaining PN-D process to allow high ${\mathrm{NH}}_{4}^{+}$-N, ${\mathrm{NO}}_{2}^{-}$-N, ${\mathrm{NO}}_{3}^{-}$-N, and COD removal in the MBR. Three phase experiments at C/N ratios of = 1.5, 2.0, and 2.5 were conducted with fixed 80 mg/L ${\mathrm{NH}}_{4}^{+}$-N to further investigate how the nitrogen and COD removal performed in the integrated MBR-MBfR system.

**Figure 5:**
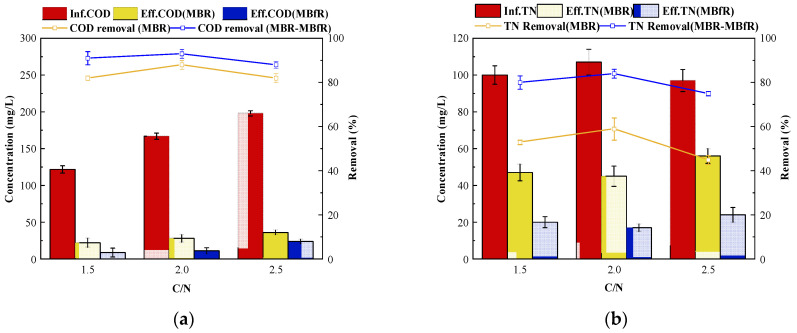

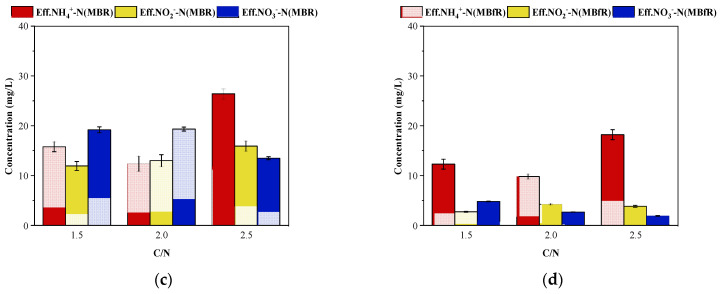
(**a**) COD removal in the MBR-MBfR during batch experiments at C/N ratios of 1.5, 2.0, and 2.5; (**b**) TN removal in the MBR-MBfR at C/N ratios of 1.5, 2.0, and 2.5; (**c**) ${\mathrm{NH}}_{4}^{+}$-N, ${\mathrm{NO}}_{3}^{-}$-N, and ${\mathrm{NO}}_{2}^{-}$-N removal of the MBR in the MBR-MBfR at C/N ratios of 1.5, 2.0, and 2.5; (**d**) ${\mathrm{NH}}_{4}^{+}$-N, ${\mathrm{NO}}_{3}^{-}$-N, and ${\mathrm{NO}}_{2}^{-}$-N removal of the MBfR in the MBR-MBfR at C/N ratios of 1.5, 2.0, and 2.5.

COD removal had no distinct change in the MBR when the C/N ratio increased from 1.5 to 2.5, as shown in Figure 5a. However, a significant decrease was found at a C/N ratio higher than two after the treatment of MBfR, owing to the increase of the influent COD concentration to 200 mg/L. The effluent COD concentrations of 8.64, 11.23, and 23.77 mg/L were detected at C/N ratios of 1.5, 2.0, and 2.5, respectively. The removal of TN in the integrated MBR-MBfR system is shown in Figure 5b. The lowest effluent TN concentration of 16.69 mg/L was detected at the influent TN concentration of 107.98 mg/L. MBfR had no significant difference in contribution to TN and COD removal among the three phase experiments at different C/N ratios.

The performance for nitrogen removal for ${\mathrm{NH}}_{4}^{+}$-N, ${\mathrm{NO}}_{3}^{-}$-N, and ${\mathrm{NO}}_{2}^{-}$-N in the MBR is shown in Figure 5c. The effluent concentrations of TN in the MBR were somewhat lower in phase I than phases II and III, which indicated that PN-D activity was greater in phase II. A dramatic increase of ${\mathrm{NH}}_{4}^{+}$-N concentration in the effluent of MBR was found owing to the suppression of the growth of AOB bacteria proliferation by organic matter, and other reason is that organic matter will be absorbed by heterotrophic aerobic bacteria in large quantities, which will inhibit the growth of AOB to a certain extent. This increase is shown in Figure 5b compared with the other two phases when the C/N ratio increased to 2.5.

In addition, the effluent ${\mathrm{NO}}_{3}^{-}$-N and ${\mathrm{NO}}_{2}^{-}$-N concentrations of the MBR were lower and higher, respectively, in the third phase because of a higher COD loading that may result in partial denitrification. The PN-D activity can often be outcompeted by heterotrophic denitrification and severely inhibited at a C/N ratio greater than 2.0.

MBfR performed an efficient removal for both nitrate and nitrite that remained in the effluent of MBR in all three phases, as shown in Figure 5d. Less than 1.90 mg/L ${\mathrm{NO}}_{3}^{-}$-N and 3.83 mg/L ${\mathrm{NO}}_{2}^{-}$-N were detected in the effluent. However, there were small but insignificant differences of ${\mathrm{NH}}_{4}^{+}$-N being reduced after the treatment of MBfR in all three phases. To our knowledge, there is little evidence in the literature that the anaerobic MBfR can effectively remove ammonium. The main contribution to TN concentration in the effluent was, therefore, that ammonium remained after the treatment of PN-D in the MBR. Therefore, in further studies, the main measure to promote TN removal could be to create a suitable condition to proliferate AOB bacteria and suppress the activity of NOB in the PN-D MBR.

### 3.4. Experimental Study on Treatment of Low C/N Wastewater by MBR-MBfR Reactor

**Figure 6:**
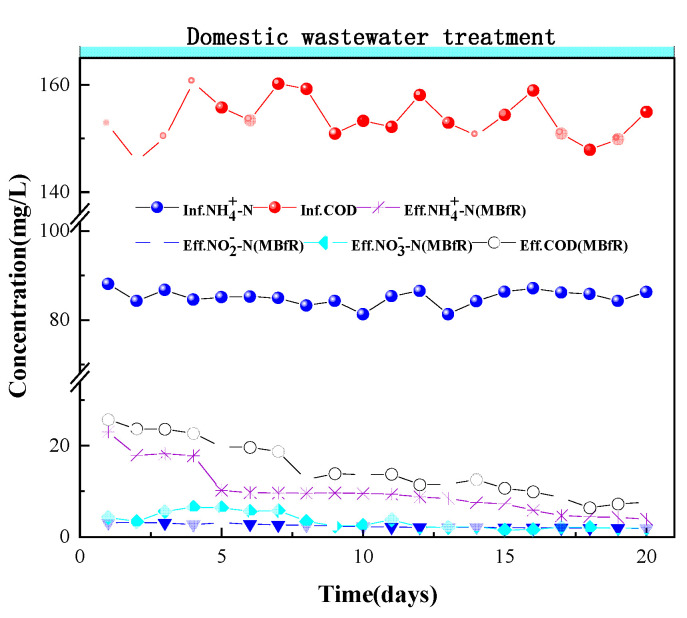
${\mathrm{NH}}_{4}^{+}$-N, ${\mathrm{NO}}_{3}^{-}$-N, ${\mathrm{NO}}_{2}^{-}$-N, and COD removal in the MBR-MBfR during the treatment and application stage.

In the treatment and application stage, the domestic wastewater came from the sewage treatment plant of Guilin University of Technology. The typical characteristics of this domestic wastewater are described in Section 2.1. After the stable operation of the reactor in the previous stage, the operation cycle was selected as 20 days. The operation results were shown in Figure 6.

At the beginning, the influent water of the MBR-MBfR changed for domestic wastewater under conditions of low DO and low C/N. The PN-D bacteria in the MBR reactor and the hydrogen autotrophic denitrifying bacteria in the MBfR reactor were affected by actual sewage. The start-up period was used for adaptation of the biomass and the PN-D process was targeted for treatment with the hydrogen autotrophic denitrification process. A short-term decline in AOB activity made the change in removal rate of COD and nitrogen not obvious and ${\mathrm{NH}}_{4}^{+}$-N built up in the effluent in the MBR at the beginning.

In the first four days, a period of acclimation for the concentration of ${\mathrm{NH}}_{4}^{+}$-N and COD was observed in the MBR-MBfR effluent. The concentration of ${\mathrm{NH}}_{4}^{+}$-N and COD decreased from 23.00 mg/L and 25.66 mg/L to 17.80 mg/L and 22.69 mg/L, respectively. The influent ${\mathrm{NH}}_{4}^{+}$-N concentration was maintained at about 80 mg/L from the fifth day to the end of the period of the domestic wastewater treatment. The effluent concentration of ${\mathrm{NH}}_{4}^{+}$-N decreased significantly from 10.21 mg/L to 3.89 mg/L compared with the first four days and the average removal rate was 90.26%. The effluent concentrations of ${\mathrm{NO}}_{3}^{-}$-N and ${\mathrm{NO}}_{2}^{-}$-N were 1.95 mg/L and 1.91 mg/L, respectively.

The steady state was reached during days 16–20. Reactor and effluent concentrations of ${\mathrm{NH}}_{4}^{+}$-N, ${\mathrm{NO}}_{3}^{-}$-N, and ${\mathrm{NO}}_{2}^{-}$-N were 4.3 ± 0.5 mg/L, 1.95 ± 0.04 mg/L, and 2.05 ± 0.15 mg/L, respectively. Here we compare the results of the proposed method with the previous study about an experiment of using a membrane bioreactor to treat actual wastewater; when the nitrogen loading rate was similar to that reported before, MBR-MBfR system could remove the excess NO_2_^-^-N and NO_3_^-^-N remaining after the partial nitrification-denitrification process, and the total nitrogen removal rate and COD removal rate could reach 84.75% and 90.57%, higher than the 43% and 87% mentioned in the previous study [27]. Compared with another study on the treatment of municipal wastewater with low C/N ratios by the A^2^O-MBR process, the total nitrogen removal rate of the MBfR in this study was greater than the 79% mentioned in previous studies (under conditions of MLSS = 3000 mg/L, close to the actual wastewater MLSS in this study) [28]. The DO concentration was at a low level between 0.4 and 0.8 mg/L. Under these conditions, the activity of AOB was enhanced and completely outcompeted NOB from the reactor. This result indicated that the PN-D process and hydrogen autotrophic denitrification in the MBR were the main processes taking place. NOB inhibition was effective while maximizing the activity of AOB, although some residual nitrite oxidation was still present. The effluent water of the MBfR met Class 1A level for Chinese discharge standards from municipal wastewater treatment plant (GB18918-2002) for ${\mathrm{NH}}_{4}^{+}$-N, ${\mathrm{NO}}_{3}^{-}$-N, and ${\mathrm{NO}}_{2}^{-}$-N concentrations.

### 3.5. Microbial Community Analysis in Different Phases of MBR-MBfR

**Figure 7:**
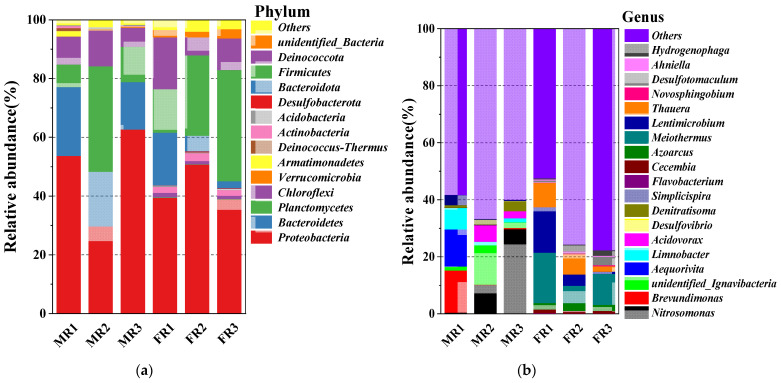
(**a**) Relative abundance of the phyla found in samples from the reactors MBR-MBfR; (**b**) relative abundance of genera found in samples from the reactors MBR-MBfR.

Sludge samples were collected on MR1 (the start-up period of MBR), MR2 (treatment stage of low C/N wastewater of MBR), MR3 (treatment stage of domestic wastewater of MBR), FR1 (the start-up period of MBfR), FR2 (treatment stage of low C/N wastewater of MBfR), and FR3 (treatment stage of domestic wastewater of MBfR). In the taxonomic analyses, the samples collected from MBR (MR1, MR2, and MR3) were grouped into 304, 324, and 363 OTUs, and the samples collected from MBfR (FR1, FR2, and FR3) were grouped into 613, 619, and 684 OTUs, respectively. The genera and phyla with relative abundance rates greater than 0.1% are shown in Figure 7.

In the sample from MBR (MR1), nine phyla with relative abundance greater than 0.1% were detected. Proteobacteria (53.54%), Bacteroidetes (23.49%), Planctomycetes (7.68%), and Chloroflexi (9.56%) were the dominant phyla in the resultant bacterial community. Proteobacteria were the predominant phyla with an occurrence of 53.54%. The genus *Brevundimonas* within this phylum had the greatest relative abundance of 14.80%. It has been reported to have a process of promoting ammonia oxidation [29] and boosting the development of the PN-D process [30]. The denitrifying bacteria of the genus *Denitratisoma*, which perform denitrification via nitrite, were present in the MR1, MR2, and MR3, with 0.80%, 1.84%, and 3.63% relative abundance. It should be noted that the main reason for the *Denitratisoma* abundance increase was that MBR-MBfR had a denitrification process, and the denitrification process was gradually enhanced after incubation with nitrogen wastewater containing carbon sources. High-throughput analysis revealed that an abundance of the genera *Denitratisoma*, which are potential denitrifiers, improved TN removal efficiency. The continuous increase of the relative abundance of *Denitratisoma* was closely related to the COD concentration rising from MR1 to MR3. This result was consistent with the findings of Ge [31] and Tao [32]. The genus *Nitrosomonas*, which belongs to the AOB, has been reported to be the first step in partial nitrification, and also found in diverse aquatic and terrestrial environments [33]. Proteobacteria and *Nitrosomonas* were the dominant phyla and genus, respectively, for MR1 to MR3. The Proteobacteria increased to 62.56% in MR3; *Nitrosomonas* increased from 0.38% to 29.53% and performed ammonium oxidation to nitrite. In the case of low DO, the NOB activity was inhibited. DO correlated with AOB and NOB abundance [34].

The abundance of partial nitrification microorganisms (*Nitrosomonas* bacteria) and denitrifying microorganisms (*Denitratisoma* bacteria) in the MBR shed light on their remarkable performance in the combined partial oxidation of ammonium and the denitrification of nitrite and nitrate. In the MBR, the species richness increased with the three phases of operation of MBR (MR1, MR2, and MR3), as evidenced by the OTUs and the Chao1 indexes. This increase was possibly because of the change from synthetic wastewater to domestic wastewater that contained complex organic matter and nitrogen compounds. The data summarized above clearly showed that partial nitrifying bacteria and denitrifying bacteria were simultaneously present in the MBR, which further demonstrates the fusion of the PN-D process in the MBR.

In the MBfR, the abundance of the ten most found bacteria at the genus and phylum level were investigated in the different phases (FR1, FR2, and FR3). The qualified sequence reads of the biological samples (FR1, FR2, and FR3) were 83270, 90419, and 80113. The most abundant genera of the operation for FR1 were *Meiothermus* and *Lentimicrobium* with a relative abundance of 17.69% and 15.96%, respectively, as shown in Figure 7b. *Meiothermus*, which has been reported as a denitrifying bacteria, played an important role of reducing Cr (VI), ${\mathrm{BrO}}_{3}^{-}$, and ${\mathrm{NO}}_{3}^{-}$ in several MBfRs [35,36]. The genus *Lentimicrobium,* known as a potential denitrifier [37], has been reported to be indispensable for the successive removal of high concentrations of nitrate [38]. The genus *Thauera*, with a relative abundance of 8.4% in MR1, could also not be ignored. *Thauera* was deemed to be the most active denitrifying bacteria in a sewage treatment system and was the most dominant and major contributor to the denitrification of nitrogen wastewater [39]. The occurrence of the genus *Hydrogenophaga* and *Desulfotomaculum* at the beginning of the operation (FR1) was 0.4% and 0.5%, respectively. They occurred as smaller populations, increasing to 1.8% and 3%, respectively, at the end (FR3). *Hydrogenophaga*, an autotrophic denitrifier, belongs to the autotrophic genera. It was a known genus of hydrogen-oxidizing bacteria dominant in the microbial community of MBfR [40]. *Hydrogenophaga* had the characteristic of dominating the biofilm and was responsible for the reduction of ${\mathrm{NO}}_{3}^{-}$ [41]. The population of *Hydrogenophaga* exhibited low relative abundances in our study compared with previous work [42,43]. The possible explanations for the lack of *Hydrogenophaga* in the present study are as follows: (1) the successful PN-D process brings about the accumulation of ${\mathrm{NO}}_{2}^{-}$ in the MBR effluent; the presence of ${\mathrm{NO}}_{2}^{-}$ has a toxic effect on *Hydrogenophaga*, and the reproduction of *Hydrogenophaga* is sensitive to its presence [17]. (2) The MBfR influent contained synthetic wastewater and domestic wastewater with COD, ${\mathrm{NO}}_{3}^{-}$, and ${\mathrm{NO}}_{2}^{-}$, and other nitrogen compounds. The loaded influent and the components were different in the MBR of each phase from FR1–FR3, which led to the limitation of the activity of *Hydrogenophaga*. Additionally, in the treatment and application stage, the treated water after the partial nitrification-denitrification process was MBfR influent, which had a more complex composition and was quite different from the experimental water in the previous study (synthetic groundwater with additives) [43]. The influent of MBfR contained a small amount of organic matter from the MBR reactor, and the presence of a small amount of residual organic matter promoted the proliferation of heterotrophic denitrifying bacteria and competed with hydrogen autotrophic denitrifying bacteria (living space and nutrients). As a result, the dominant species of hydrogen autotrophic denitrifying bacteria possessed a low stable relative abundance for a long time.

Meanwhile, the entry of organic matter in the influent made heterotrophic denitrifying bacteria compete more effectively with the autotrophic denitrifying bacteria. The heterotrophic denitrifying bacteria therefore had an advantage, seizing the electron donor, which resulted in inhibition of the growth of the autotrophic denitrifying bacteria.

## 4. Conclusions

A two-stage system was applied for nitrogen removal from a wastewater treatment plant processing wastewater in an MBR-MBfR reactor. The proper functioning of the system was achieved by coupling the PN-D process in an MBR with further treatment in an MBfR. More than 96% of ${\mathrm{NH}}_{4}^{+}$-N was removed via PN-D in MBR. In experiments with C/N (ratios of 1.5, 2.0, and 2.5) for the MBR-MBfR, the PN-D process was often outcompeted by heterotrophic denitrification and severely inhibited at a C/N greater than 2.0. MBfR performed a further treatment for both nitrate and nitrite that remained in the effluent of MBR, which obtained the average ${\mathrm{NO}}_{3}^{-}$-N removal of 89.3% when the influent ${\mathrm{NO}}_{3}^{-}$-N concentration was 30 mg/L and the HRT was 5 h. The effluent water of MBfR met Class 1A level for Chinese discharge standards after the stable operation of the MBR-MBfR (in the experimental conditions of HRT = 15 h, SRT = 20 d) used for domestic wastewater treatment. Microbial community analysis revealed that a successful AOB-proliferation stage was achieved with denitrifying bacteria (*Denitratisoma* genus), which performed denitrification in MBR at the same time. In the MBfR, the dominant bacteria were *Meiothermus*, *Lentimicrobium*, *Thauera*, *Hydrogenophaga,* and *Desulfotomaculum*, which proved the success of the hydrogenotrophic denitrification process in MBfR and showed the characteristics of efficient nitrate and nitrite removal.

## Figures and Tables

**Table 1:** The physical characteristics of MBR-MBfR.

Reactor	Parameter	Units	Value
MBR	MBR height	cm	30
	Number of HFM		80
	HFM inner diameter	mm	1.2
	HFM outer diameter	mm	2.2
	HFM pore size	μm	0.1
	Active surface area	m^2^	0.06
	Active volume	L	4.32
MBfR	MBfR height	cm	64
	Number of HFM		65
	HFM inner diameter	mm	1.0
	HFM outer diameter	mm	1.66
	HFM pore size	μm	0.02
	Active surface area	m^2^	0.28
	Active volume	L	1.8

**Table 2:** Operational conditions during the start-up of partial nitrification in the MBR.

Phase	Time (days)	${\mathrm{NH}}_{4}^{+}$-N (mg/L)	${\mathrm{NO}}_{3}^{-}$-N (mg/L)	HRT (h)
I	1–7	50.11	10.90	16
II	8–15	61.66	10.10	14
III	16–23	71.43	9.41	12
IV	24–30	80.74	9.90	10

**Table 3:** Operational conditions during the different phases with various C/N ratios in the MBR.

Phase	Time (Days)	C/N	COD (mg/L)	${\mathrm{NH}}_{4}^{+}$-N (mg/L)	HRT (h)
I	31–38	0.5	40	80	10
II	39–46	1	80		
III	47–54	2	160		
IV	55–62	3	240		

**Table 4:** Operational conditions at various C/N ratios in the MBR.

Phase	C/N	COD (mg/L)	${\mathrm{NH}}_{4}^{+}$-N (mg/L)	HRT (h)
I	1.5	120	80	15
II	2.0	160		
III	2.5	200		

## Data Availability

The data that support the findings of this study are available from the corresponding author upon reasonable request.
